# Genetic background of primary and familial HLH in Qatar: registry data and population study

**DOI:** 10.3389/fped.2024.1326489

**Published:** 2024-05-09

**Authors:** Elkhansa Elgaali, Massimo Mezzavilla, Ikhlak Ahmed, Mohammed Elanbari, Aesha Ali, Ghada Abdelaziz, Khalid A. Fakhro, Ayman Saleh, Tawfeg Ben-Omran, Naima Almulla, Chiara Cugno

**Affiliations:** ^1^ Pediatric Hematology and Oncology Department, Sidra Medicine, Doha, Qatar; ^2^ Department of Biology, University of Padua, Padua, Italy; ^3^ Research Department, Sidra Medicine, Doha, Qatar; ^4^ Division of Pediatrics, Sidra Medicine, Doha, Qatar; ^5^ Division of Genetic and Genomic Medicine, Sidra Medicine, Doha, Qatar; ^6^ Department of Medical Genetics, Hamad Medical Corporation, Doha, Qatar

**Keywords:** familial hemophagocytic lymphohistiocytosis (FHLH), genetics, novel variants, QGP, hemophagocytic lymphohistiocytosis (HLH)

## Abstract

**Background:**

Familial hemophagocytic lymphohistiocytosis (FHLH) is an inherited life-threatening disease. Five types are identified, with the addition of congenital immunodeficiency syndromes in which HLH is a typical manifestation. The literature on this disease is very scarce in the Middle East, with only a few scattered reports.

**Methods:**

We report detailed demographic, clinical, and genomic data from 28 patients diagnosed with primary and familial HLH over the last decade in Qatar. An evaluation was performed of allele frequencies of deleterious variants from 12 primary and familial HLH causative genes on the Qatar Genome Programme (QGP) cohort of 14,669 Qatari individuals.

**Results:**

The genetic diagnosis was obtained in 15 patients, and four novel mutations in *Perforin 1* (*PRF1*), *UNC13D*, *LYST*, and *RAB27A* genes were found. We identified 22,945 low/high/moderate/modifier impact variants significantly enriched in the QGP in those 12 genes. The variants rs1271079313 in *PRF1* and rs753966933 in *RAB27A* found in our patient cohort were significantly more prevalent in the QGP compared to the Genome Aggregation Database (gnomAD) database, with a high carrier frequency in the Qatari population.

**Conclusions:**

We established the first primary and familial HLH Registry in the Gulf Region and identified novel possibly pathogenic variants present at higher frequency in the Qatari population, which could be used for screening purposes. Raising awareness about primary and familial HLH and implementing screening activities in the Qatari highly inbred population could stem into more comprehensive premarital and prenatal evaluations and faster diagnosis.

## Introduction

Hemophagocytic lymphohistiocytosis (HLH) represents a hyperinflammatory syndrome characterized by reduced cytotoxic T-cell and natural killer (NK)-cell activity and uncontrolled aggressive proliferation of cytotoxic lymphocytes, leading to increasing levels of activated macrophages and inflammatory cytokines, which target multiple organs including the spleen, liver, bone marrow, and brain (1).

HLH is classified into primary, familial (FHLH), and secondary HLH. FHLH is an autosomal recessive rare immunodeficiency with onset in early childhood defined by mutations in FHL-causing loci, which render the cytotoxicity pathway ineffective in eradicating immune stimuli. Five types are identified, with four of the gene mutations described and discussed in the literature. Locus 9q21.3–22 has been associated with FHLH type 1 [Online Mendelian Inheritance in Man (OMIM) #267700]; however, the corresponding gene defect and protein have not been recognized so far. FHLH types 2–5 are caused by mutations in the perforin (*PRF1*, OMIM #603553), *UNC13D* (OMIM #608898), Syntaxin 11 (*STX11*, OMIM #603552), and syntaxin-binding protein-2 (*STXBP2*, OMIM #613101) genes (2, 3).

Additional genetic diseases in which HLH is a typical and common manifestation (primary HLH) include congenital immunodeficiency syndromes, such as Griscelli syndrome type 2 (GS2), caused by mutations of *RAB27A*, Chédiak–Higashi syndrome, X-linked lymphoproliferative disease, X-linked immunodeficiency with magnesium defect, interleukin-2-inducible T-cell kinase deficiency, and Hermansky–Pudlak syndrome. Acquired or secondary HLH can be triggered by infections, neoplasia, and autoimmune inflammatory syndromes, or can be found in the context of metabolic disorders and immune deficiencies, such as CD27 deficiency, lysinuric protein intolerance, chronic granulomatous disease, and so on (4).

The onset of the clinical manifestation of primary and familial HLH usually occurs in the first year of life, with the disease developing between 1 and 6 months of age or even *in utero* and manifesting at birth, and it is invariably fatal if left untreated. The common clinical findings include fever, hepatosplenomegaly, and bicytopenia (affecting at least two of three lineages in the peripheral blood: erythrocytes, leukocytes, and thrombocytes). Neurologic abnormalities can be isolated or indicate advanced primary and familial HLH. The diagnosis of primary and familial HLH is made by either fulfilling the HLH-2004 protocol's clinical and laboratory diagnostic criteria, or genetic testing that reveals an HLH-causing mutation (5). Early diagnosis and prompt and aggressive initiation of therapy can be life-saving (5).

To our knowledge, the literature on primary and familial HLH is very scarce in the Middle East and North Africa. Reports from Israel (6) and Oman (7) have described the clinical presentation and outcome of 11 and 13 children diagnosed with FHLH, respectively. The largest case series was recently published and reported 87 Saudi patients diagnosed with both familial and secondary HLH between 1995 and 2014: 36% of FHLH patients were found to have a *STXBP2* mutation (8). In another case series, five patients with *STX11* mutation and two patients with *PRF1* mutation were described in a highly consanguineous setting (9).

GS2 (OMIM: 607624), caused by pathogenic variants in the *RAB27A* gene, is characterized by partial albinism, variable T lymphocyte and NK-cell deficiencies, and neurological impairment, i.e., seizures, strabismus, hemiparesis, ataxia, and cognitive disorders (10–12).

Al-Sulaiman et al. recently described a founder *RAB27A* variant (NM_183235.3:c.244C>T) causing GS2 in highly consanguineous Qatari families (10). HLH was diagnosed in 33% of the patients and suspected in an additional 33% of patients who died. Overall, a relevant phenotypic heterogeneity was found, ranging from asymptomatic individuals to patients with isolated skin manifestations or neurological complications and/or HLH (10).

The aims of the present study were as follows: (1) to report on primary and familial HLH cases in Qatar in the last decade, while establishing the first Registry in the Gulf Region; and (2) to investigate the genetic background of the primary and familial HLH spectrum in the Qatari population, exploring the allele frequency of the variants in known HLH causative genes in the context of the Qatar Genome Programme (QGP), which is a population genome project aiming to sequence the genomes of the local population (13).

## Methods

Detailed demographic, clinical, and genomic data were retrospectively collected from 28 patients diagnosed with primary and familial HLH between 2010 and 2022 at the Pediatric Oncology and Hematology, and Immunology Departments of Hamad General Hospital and Sidra Medicine (Doha), which is the only tertiary pediatric care hospital in Qatar. The clinical diagnosis was made in the presence of molecular confirmation or meeting five of the following eight criteria: fever; splenomegaly; cytopenia affecting at least two out of three peripheral blood lineages; hypertriglyceridemia and/or hypofibrinogenemia; hemophagocytosis in bone marrow, spleen, or lymph nodes; low or absent NK-cell activity; hyperferritinemia; or high soluble CD25/interleukin-2 receptor levels.

A total of 15 patients affected by GS2 were previously partially reported (10).

Clinical exome sequencing was performed on 24 patients at GeneDx, USA, as previously described (10). Four patients (patients 3, 4, 6, and 10) received diagnoses based solely on clinical criteria and did not undergo subsequent genetic testing confirmation, as detailed in Table 1.

**Table 1:** Clinical features, laboratory characteristics, and genetic findings of the patients’ cohort.

Patient code	Ethnicity	Age at diagnosis (months)	Gender	Cytopenia >2 lines	Ferritin (mg/L)	Diagnostic criteria (out of 8)	CNS involvement	Causative gene	Variant	Genotype	Previously described or novel	HSCT	Outcome
FHLH													
PT#1	Arab-Qatari	3	Female	Yes	>7,500	5	No	*PRF1*	NM_001083116.3:c.50del	Homozygous	Previously described (14–22)	Yes	Alive
PT#2	Arab-Qatari	1	Female	Yes	>7,500	5	No	*PRF1*	NM_001083116.3:c.893A>G	Homozygous	Novel	Yes	Alive
PT#3	Asian	79	Male	Yes	21,225	6	No	NA	NA	NA	NA	No	Dead
PT#4	Asian	116	Female	No	328	5	No	NA	NA	NA	NA	No	Alive
PT#5	Asian	4	Female	Yes	2,139	6	NA	*UNC13D*	NM_199242.3:c2955-20_2955-1del	Homozygous	Novel	Yes	Alive
PT#6	Asian	36	Female	Yes	40,000	5	No	NA	NA	NA	NA	NA	Alive
PT#7	Arab non-Qatari	3	Female	Yes	16,930	6	No	*PRF1*	NM_001083116.3:c.658G>A	Homozygous	Previously described (21)	No	Dead
PT#8	Arab non-Qatari	88	Male	No	69	CNS only	Yes	*PRF1*	NM_001083116.3.c.673C>T	Homozygous	Previously described (15, 17, 22–27)	NA	NA
PT#9	Asian	6	Female	Yes	1,853	6	No	*UNC13D*	NM_199242.3:c2955-20_2955-1del	Homozygous	Novel	No	Dead
PT#10	Arab non-Qatari	1.5	Male	Yes	>7,500	5	No	NA	NA	NA	NA	No	Dead
PT#11	Caucasian	1.5	Female	Yes	3,671	6	Yes	*UNC13D*	NM_199242.3:c.2346_2349del	Homozygous	Previously described (20, 21, 27–33)	Yes	Dead
PT#12	Asian	2	Female	Yes	1,388	7	Yes	*LYST*	NM_000081.4.c281C>T	Heterozygous	Novel	No	Alive
PT#13	Arab non-Qatari	66	Female	Yes	1,421	5	Yes	*PRF1*	NM_001083116.3(PRF1):c.133G>A NM_001083116.3(PRF1):c.148G>A	Heterozygous Heterozygous	Previously described (15, 20, 34–36) Previously described (15, 20, 23, 37–40)	Yes	Alive
HLH in Griscelli type 2													
PT#14	Arab non-Qatari	36	Female	No	NA	1	Yes	*RAB27A*	NM_183235.3:c.400A>G	Homozygous	Novel	No	Dead
PT#15	Arab non-Qatari	108	Male	Yes	NA	3	No	*RAB27A*	NM_183235.3:c.400A>G	Homozygous	Novel	No	Dead
PT#16	Arab non-Qatari	28	Female	No	63	1	Yes	*RAB27A*	NM_183235.3:c.550C>T	Homozygous	Previously described (20, 21, 41–47)	No	Dead
PT#17	Arab-Qatari	114	Male	Yes	153	4	Yes	*RAB27A*	NM_183235.3:c.244C>T	Homozygous	Previously described (10, 20, 21, 35, 48–52)	Yes	Alive
PT#18	Arab-Qatari	48	Male	NA	NA	CNS only	Yes	*RAB27A*	NM_183235.3:c.244C>T	Homozygous	Previously described (10, 20, 21, 35, 48–52)	Yes	Alive
PT#19	Arab-Qatari	204	Male	No	14	2	No	*RAB27A*	NM_183235.3:c.244C>T	Homozygous	Previously described (10, 20, 21, 35, 48–52)	No	Alive
Griscelli type 2 at risk for HLH													
PT#20	Arab-Qatari		Male	N/A	N/A	N/A	No	*RAB27A*	NM_183235.3:c.244C>T	Homozygous	Previously described (10, 20, 21, 35, 48–52)	Yes	Alive
PT#21	Arab-Qatari		Male	N/A	N/A	N/A	No	*RAB27A*	NM_183235.3:c.244C>T	Homozygous	Previously described (10, 20, 21, 35, 48–52)	No	Alive
PT#22	Arab-Qatari		Male	N/A	N/A	N/A	No	*RAB27A*	NM_183235.3:c.244C>T	Homozygous	Previously described (10, 20, 21, 35, 48–52)	No	Alive
PT#23	Arab-Qatari		Male	N/A	N/A	N/A	No	*RAB27A*	NM_183235.3:c.244C>T	Homozygous	Previously described (10, 20, 21, 35, 48–52)	Yes	Alive
PT#24	Arab-Qatari		Female	N/A	N/A	N/A	No	*RAB27A*	NM_183235.3:c.244C>T	Homozygous	Previously described (10, 20, 21, 35, 48–52)	No	Alive
PT#25	Arab-Qatari		Female	N/A	N/A	N/A	No	*RAB27A*	NM_183235.3:c.244C>T	Homozygous	Previously described (10, 20, 21, 35, 48–52)	No	Alive
PT#26	Arab-Qatari		Female	N/A	N/A	N/A	No	*RAB27A*	NM_183235.3:c.244C>T	Homozygous	Previously described (10, 20, 21, 35, 48–52)	No	Alive
PT#27	Arab-Qatari		Male	N/A	N/A	N/A	No	*RAB27A*	NM_183235.3:c.244C>T	Homozygous	Previously described (10, 20, 21, 35, 48–52)	No	Alive
PT#28	Arab non-Qatari		Female	N/A	N/A	N/A	No	*RAB27A*	NM_183235.3:c.400A>G	Homozygous	Previously described (53)	No	Alive

NA, not available.

This Registry study was conducted under the ethical approval of Sidra Medicine Institutional Review Board (IRB) (protocol no. 1760852).

The QGP study population consisted of 14,669 apparently healthy adult Qatari individuals who gave consent and were recruited by the Qatar Biobank (QBB) (13), and who underwent whole-genome sequencing at Sidra Medicine.

In the QGP study, only anonymized datasets were accessed and used for the analysis after obtaining approval from the QBB IRB (QF-QBB-RES-ACC-00052).

### Whole-genome sequencing data

The evaluation of allele frequency of primary and familial HLH causative genes (*PRF1*, *UNC13D*, *STX11*, *STXBP2*, *SH2D1A*, *XIAP*, *RAB27A*, *LYST*, *AP3B1*, *ITK*, *MAGT1*, and *NLRC4*) was performed on the QGP cohort: raw data generated from those samples were mapped to the human reference genome (genome reference consortium human build 38 patch, GRCh38) using a Burrow-Wheeler Aligner (BWA). Joint variant calling was performed using the Genome Analysis Toolkit (GATK). Quality control (QC) was performed at different stages of the pipeline to ensure data quality and consistency. Poor quality samples were removed based on the low call rate due to poor DNA quality, outlying heterozygosity due to sample contamination or inbreeding, duplication or relatedness of samples based on identity by state (IBS), mismatches with external information, and outlying population ancestry due to population structure. Variant quality control was performed as previously described (54). The specific set of variants encompassing primary and familial HLH causative genes was extracted from the multi-sample vcf file using bcftools (55) for interrogation of their pathogenic effects in our population. Genetic variations from those genes were extracted from the sequencing data and annotated using SnpEff/SnpSift (v4.3t) (https://pcingola.github.io/SnpEff/), categorizing their impact on gene function as “high,” “moderate,” “low,” and “modifier.”

### Statistical analysis

Fisher's exact test was applied to identify variants in the 12 known primary and familial HLH causative genes that showed a statistically different enrichment of the alternate allele frequency between QGP and the Genome Aggregation Database (gnomAD) release 3.0.

We corrected for multiple testing using the Benjamini–Hochberg method for controlling the false discovery rate (FDR). All variants with an adjusted *p*-value (adjusted) <0.05 were considered to have differential enrichment.

Analyses were run using R software version 4.0.4.

In addition, we analyzed the level of genic constraints for each primary and familial HLH gene using two metrics: (1) the loss of function intolerance score (pLi) (56), measuring from gnomAD how much a gene is prone to accumulate loss of function variants; and (2) the residual variation intolerance score (RVIS) (57), indicating whether a gene has more or less common functional genetic variation compared to the genome-wide expectation. A relatively essential or fundamental gene should have a high intolerance of both loss of function and functional variation (high pLi, low RVIS). Finally, we explored the possible protein–protein interactions (PPIs) between primary and familial HLH genes using the data obtained from the BioGRID database (https://thebiogrid.org/) (58).

## Results

Based on HLH-2004 criteria, a total of 19 out of 28 (67.8%) patients were diagnosed with primary and familial HLH, 6 (21.4%) of them in the context of GS2. The detailed clinical data of the patients are reported in Table 1.

The patients (63.2% female; 36.8% male) had a mean age at the time of diagnosis of 31.3 and 89.7 months, respectively for isolated FHLH and primary HLH with underlying GS2. Different ethnicities were represented: Qatari (*n* = 5, 26.3%), other non-Qatari Arabs (*n* = 7, 36.8%), Asian (*n* = 6, 31.7%), and Caucasian (*n* = 1, 5.2%).

Primary and familial HLH were localized only to the central nervous system (CNS) in five (26.3%) patients (four in the GS2 group). Overall, CNS involvement (clinical symptoms and/or radiological findings and/or cerebrospinal fluid pleocytosis/hemophagocytosis) was identified in eight (44.4%) patients.

Other clinical diagnostic criteria were fever (83.3%); splenomegaly (74%; spleen >3 cm below costal margin); cytopenia >2 lineages (72.2%; hemoglobin <9 g/dl, absolute neutrophil count <100/µl, platelets < 100,000/µl); hypertriglyceridemia and/or hypofibrinogenemia (68.7%); hemophagocytosis in bone marrow, spleen, or lymph nodes (43.7%); hyperferritinemia (68.7%; >500 mg/L); and high soluble CD25/interleukin-2 receptor levels (100%; available only for eight patients). An NK cytotoxicity test was not performed due to the lack of testing availability in Qatar.

Genetic studies were performed in 15 patients and the following genes were identified: *PRF1* (33.3%), *UNC13D* (20%), and *RAB27A* (40%). *LYST* was detected in only one (6.6%) patient. We found four novel mutations not previously described in *PRF1*, *UNC13D*, *LYST*, and *RAB27A* (Table 1).

Two patients (patients 8 and 18) presented with isolated CNS HLH, exhibiting suggestive radiological findings alongside a positive molecular diagnosis. Isolated CNS HLH is increasingly recognized as a distinct clinical entity, characterized by chronic inflammation confined to the CNS and germline mutations in known primary HLH-associated genes (59).

We also report on 15 GS2 patients: 6 developed primary HLH, 2 of them are alive after hematopoietic stem cell transplantation (HSCT), and 1 is awaiting the transplant procedure. A total of 11 children carried the founder mutation (NM_183235.3:c.244C > T) described by Al-Sulaiman et al. (10). Three patients with different *RAB27A* mutations died before having access to HSCT. Out of the 11 GS2 patients with *RAB27A* Qatari founder mutation, 2 were pre-emptively transplanted before developing any signs or symptoms of HLH while the others are currently being closely followed.

The patients were mostly treated with HLH protocols 1994 and 2004, and 7 out of 17 underwent HSCT.

The mortality rate was 42% (one patient was lost to follow-up). Seven out of eight patients died early after the onset of the disease, while one died from HSCT-related complications. The mortality rate for GS2 patients with primary HLH was 50%.

In the QGP cohort, only three variants (rs147035858, rs1271079313, and rs753966933), all identified in Qatari patients, were found. The variants rs1271079313 and rs753966933 were significantly more frequent in QGP when compared to the gnomAD database. A total of 19 heterozygous carriers for rs1271079313, and 145 heterozygous carriers and 7 homozygous individuals for rs753966933 were identified in the QGP cohort (Table 2).

**Table 2:** Allele frequencies of patients’ variants in Qatar Genome Programme and gnomAD.

Gene	Variant	ID	Region	gnomAD Freq	QGP Freq	Hom. individuals in QGP	Het. Individuals in QGP	adj-*p-*value
*PRF1*	NM_001083116.1:c.50de	rs147035858	Qatari	C = 0.0002371	C = 0.000238598	—	—	1
*PRF1*	NM_001083116.3:c.893A>G	rs1271079313	Qatari	C = 0.00000795	C = 0.000647624	—	19	1.13E-16
*PRF1*	NM_001083116.3:c.658G>A	rs776571416	Arab non-Qatari	T = 0.000007	Not present	—	—	NA
*PRF1*	NM_001083116.3.c.673C>T	rs28933973	Arab non-Qatari	A = 0.000012	Not present	—	—	NA
*UNC13D*	NM_199242.3:c.2955-5C>T	rs201791093	Asian	A = 0.000013 (deletion not found, only 1 SNP in this position)	Not present	—	—	NA
*UNC13D*	NM_199242.3:c.2346_2349de	rs764196809	Caucasian	delCCCT = 0.000108	Not present	—	—	NA
*LYST*	NM_000081.4:c.281C>T	rs777389303	Arab non-Qatari	A = 0.000240	Not present	—	—	NA
*RAB27A*	NM_183235.3:c.400A>G	NA	Arab non-Qatari	not present (literature only)	Not present	—	—	NA
*RAB27A*	NM_183235.3:c.550C>T	rs200956636	Arab non-Qatari	A = 0.000068	Not present	—	—	NA
*RAB27A*	NM_183235.3:c.244C>T	rs753966933	Qatari	A = 0.000016	A = 0.00541959	7	145	2.89E-148

Freq, frequency; Het, heterozygous; Hom, homozygous.

When restricting the analysis to unrelated samples (individuals not sharing a first- or second-degree relationship with any other individual in the QGP cohort), 18 primary and familial HLH gene variants were found at a higher frequency in QGP samples than in gnomAD samples (OR >1; 95% confidence interval (CI); adj-*p-*value <0.05); none of these variants was identified in our patient cohort (Supplementary Table 1).

**Figure 1:**
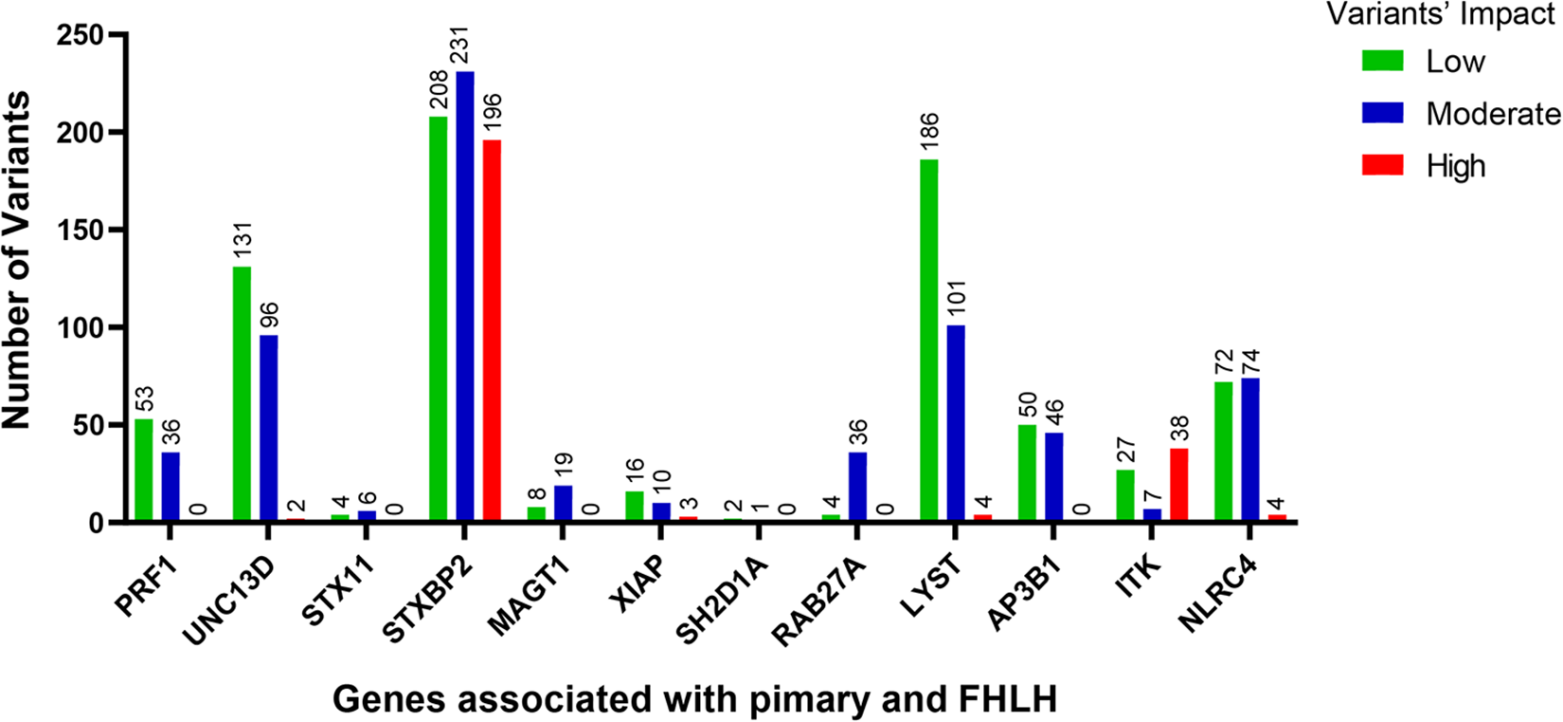
The number of variants with low (green bars), moderate (blue bars) and high (red bars) impact significantly enriched in the QGP cohort vs. gnomAD database. Fisher's exact test was carried out for comparisons between QGP and gnomAD to identify variants in the 12 known primary and FHLH causative genes. Adj-*p*-value <0.05.

The low/high/moderate impact variants significantly enriched in the QGP vs. gnomAD (adj-*p-*value <0.05) were 22,945, with higher values for *STXBP2* and *LYST* (Figure 1).

In addition, we observed that three out of four genes (*PRF1*, *LYST*, and *RAB27A*) present in our cohort harbored a high level of genic intolerance (low RVIS, <25%), whereas none of them showed evidence of pLi (Table 3).

**Table 3:** Genic intolerance scores of the 12 primary and FHLH causative genes.

Gene	pLi	RVIS	RVIS (%)
*PRF1*	0	−0.62	17.45
*UNC13D*	0	0.08	59.44
*STX11*	0	0.64	83.98
*STXBP2*	0	0.43	77.29
*SH2D1A*	0.39	0.06	58
*XIAP*	0.92	0.02	55.22
*RAB27A*	0	−0.56	19.31
*LYST*	0.02	−3.04	0.5
*AP3B1*	0.62	0.43	77.29
*ITK*	0	−0.22	37.43
*MAGT1*	0.96	0.1	61.49
*NLRC4*	0	−0.6	18.21

All primary and familial HLH genes found in our cohort did not display any evidence of physical interaction (PPI) between each other except for *RAB27A* with *UNC13D* (data not shown).

## Discussion

The present study reports all cases of primary and familial HLH diagnosed in Qatar over the last decade and establishes the first HLH Registry in the Gulf Region.

We found that the majority (72%) of the patients had been diagnosed in the last 3 years, indicating both increased awareness of the clinical team and meaningful use of next-generation sequencing techniques for the routine diagnostic workup. Signs and symptoms often appear sequentially or late during the disease course, rendering the diagnosis difficult; molecular testing has the advantage of allowing early diagnosis in suspected cases where not all required clinical criteria are yet fulfilled.

Managing isolated CNS HLH presents significant challenges due to its elevated mortality and morbidity rates. This may be due to distinct neuroinflammatory processes triggering isolated disease manifestation without systemic activation, potentially influenced by specific genetic factors (60). The patients listed in Table 1 who had only one diagnostic criterion or exhibited CNS involvement were the ones who benefited the most from the early molecular workup. This enabled a timely diagnosis before the clinical presentation could manifest in its full severity.

In our cohort of mostly Arabic and Asian patients, we described four novel mutations, previously categorized as of uncertain significance, in *PRF1* (NM_001083116.3:c.893A > G), *UNC13D* (NM_199242.3:c2955-20_2955-1del), *LYST* (NM_000081.4.c281C > T), and *RAB27A* (NM_183235.3:c.400A > G), whose recognition as disease causative will surely help to drive faster diagnosis and treatment.

In our study, we observed that certain genes related to primary and familial HLH within our patient cohort showed lower scores in a measurement called RVIS compared to other genes linked to the same disease. This suggests a high level of purifying selection among these genes, except for *UNC13D*, and explains why the deleterious variants were found mainly in *RAB27A*, *PRF1*, and *LYST*. Our findings indicate that three distinct genes within the studied cohort, namely *PRF1*, *LYST*, and *RAB27A*, manifest a heightened degree of genic intolerance (the degree to which a gene can tolerate genetic variation without adversely affecting its function), as denoted by their low RVIS (which falls under the 25th percentile of the genomic distribution of all RVIS). This should be interpreted as a reduced tolerance to genetic variations. However, noteworthy is the absence of discernible evidence denoting pronounced intolerance to loss-of-function mutations, as reflected by the lack of pLi values higher than 0.9 (61) in these three genes.

We can summarize that these genes, classified as highly sensitive to mutations and genetic changes or “mutation and/or genic intolerant” by these measures, should be enriched for variants that lead to genetic diseases compared to genes that are more tolerant to mutations and classified as “ mutation and genic tolerant” (62, 63). More precisely, these genes show evidence of intolerance to functional variation (RVIS); however, they appear to be tolerant to putative loss of function variants. An interpretation is that there are few deleterious effects if these genes are not expressed due to the presence of loss of function variants, but the presence of missense variants that alter the function of these genes is less tolerated in the population due to their possible pathogenic effects.

Although all the genes are required for the assembly, exocytosis, and function of cytotoxic granules (64), the majority of them act independently at different steps of the cytotoxic degranulation pathway, as indicated by the absence of evident protein–protein interactions in our data. The lack of interactions should rule out any hypothesis of epistatic interplay between these genes. The only exception is represented by *RAB27A* with *UNC13D*, which in fact are both involved in the docking and priming of the cytotoxic granules (64, 65).

We performed a comprehensive analysis of the variants annotated that were significantly different between the Qatari population (14,669 whole genomes) and other world populations represented in the gnomAD v3 dataset (76,156 whole genomes): 22,945 variants predicted as low/high/moderate impact in the 12 known primary and familial HLH causative genes had higher allele frequencies in the Qatari population (Figure 1), confirming recent publications showing that several rare deleterious variants are more common in the Qatari population, in line with the high consanguinity rate (54).

Two of our variants of interest (rs1271079313 and rs753966933) were found to be considerably enriched in the Qatari population. The *RAB27A* variant (rs753966933) has already been described as a founder mutation for Qatar in a recent publication (10), while the *PRF1* variant (rs1271079313) has not reported before and can be considered a population-enriched variant that drifted to an elevated allele frequency in Qatar.

The *RAB27A* variant (rs753966933) was identified in 7 homozygous Qatari individuals and 145 carriers. This possibly pathogenic variant is considered highly deleterious according to its combined annotation-dependent depletion (CADD) score (CADD = 33) (66); to our knowledge, no homozygous individuals were found in other databases, such as gnomAD and the 1000 Genomes Project (1KG). This observation, coupled with the level of constraint found in this gene, could highlight a possible increased burden of deleterious variants, due to endogamy and founder effects, which are common characteristics of the Qatar population in *RAB27A* (54), which ultimately could lead to an increased prevalence of primary HLH in this population.

No homozygous individuals were identified for the *PRF1* variant (rs1271079313) in the QGP, while 19 carriers were observed.

Our data suggest that these inherited possibly pathogenic variants should be taken into consideration for premarital or early diagnostic screenings, which are currently limited in Qatar. They might be included in the newly developed “QChip1,” a genotyping microarray including the most common single gene disease pathogenic variants identified in Qatar that could become a valuable screening tool for newborns, premarital couples, and patients (67).

We corroborated recently published data describing additional patients affected by GS2 and characterized by an extremely wide spectrum of clinical manifestations, ranging from asymptomatic individuals to life-threatening HLH (10). The penetrance of the *RAB27A* mutation (NM_183235.3:c.244C>T) remains unclear, especially considering the identification of seven homozygous, apparently healthy, individuals in the QGP. There is increasing evidence that partial and combined genetic defects of the degranulation pathway can predispose to HLH together with environmental stimulus and/or genetic modifiers (64) and that several digenic combinations for HLH are also present at some frequency in healthy populations (68). Further studies are needed to identify those individuals at high risk of developing HLH, hence benefitting from preventative treatment. The use of whole exome/genome sequencing instead of targeted sequencing/genotyping could help to better understand the combined role of different genes involved in the granulation pathway and their clinical impact.

We believe that raising awareness about this disease as well as early consideration of signs and symptoms could lead to a faster diagnosis, shorten the diagnostic odyssey, prevent fatal outcomes, and facilitate a rational therapeutic approach, especially with the advent of gene therapy and gene editing methods. It will also lead to the identification of new variants, enabling the offering of various preventative measures and genetic counseling in the premarital, preconceptional, or prenatal period.

## Data Availability

Whole-genome sequence data related to 14,669 Qatari individuals can be accessed through application to the Qatar Biobank (https://www.qatarbiobank.org.qa/) by submitting an online request, subject to institutional review board approval by the Qatar Biobank.
