# Supplementary material for: Genetic background of primary and familial HLH in Qatar: registry data and population study
Source: Front Pediatr. 2024 May 9;12:1326489. doi: 10.3389/fped.2024.1326489 (PMC11130942; doi:10.3389/fped.2024.1326489)
Supplement: Supplementary file 1 [file Table1.pdf]

SUPPLEMENTARY TABLE 1 Enriched variants in QGP unrelated samples compared to gnomAD database.

Gene	Mutation	AF (QGP UNRELATED)	AF (gnomAD)	OR	CI (lower)	CI (upper)	*P*- value	adj-*p* value
UNC13D	NM_199242.3: c.3260C>T	0.083	0.049	1.71	1.59	1.84	2.20E-43	9.45E-43
UNC13D	NM_199242.3:c.2943G>A	0.073	0.052	1.41	1.31	1.52	3.20E-18	6.26E-18
UNC13D	NM_199242.3:c.2299-46C>T	0.279	0.125	2.22	2.13	2.32	7.30E-270	8.56E-269
UNC13D	NR_199242.3: c.4916T>C	0.317	0.139	2.28	2.19	2.37	3.0E-318	4.71E-317
UNC13D	NM_199242.3: c.58C>T	0.078	0.058	1.35	1.25	1.45	2.20E-15	4.06E-15
UNC13D	NM_199242.3: c.3260C>T	0.048	0.002	29.25	25.55	33.5	0.00E+00	0.00E+00
RAB27A	NM_183235.3:c.*14C>T	0.222	0.193	1.15	1.1	1.21	8.90E-10	1.16E-09
LYST	NM_000081.4:c.10564+43G>A	0.45	0.42	1.07	1.03	1.11	1.30E-04	1.56E-04
LYST	NM_000081.4:c.5023+41C>T	0.14	0.104	1.34	1.27	1.42	1.60E-24	3.77E-24
LYST	NM_000081.4:c.4392C>T	0.125	0.088	1.42	1.34	1.5	3.40E-30	1.01E-29
LYST	NM_000081.4:c.2355T>C	0.141	0.105	1.35	1.27	1.42	3.60E-25	9.27E-25
AP3B1	NM_003664.4:c.280-28T>C	0.409	0.324	1.26	1.22	1.31	3.20E-36	1.08E-35
FAM71B	NM_130899.2:c.1691T>C	0.187	0.108	1.73	1.65	1.82	7.40E-97	3.48E-96
MED7	NM_130899.3:c.921T>C	0.23	0.126	1.83	1.75	1.91	1.20E-137	9.63E-137
ITK	NM_005546.3:c.495+22G>A	0.288	0.27	1.07	1.02	1.11	2.10E-03	2.41E-03
ITK	NM_005546.3:c.647+28C>T	0.085	0.068	1.26	1.17	1.35	2.00E-10	2.66E-10
ITK	NM_005546.3:c.1232+19G>A	0.068	0.054	1.26	1.17	1.36	5.40E-09	6.86E-09
ITK	NM_005546.3:c.1792-41T>C	0.438	0.409	1.07	1.04	1.11	9.20E-05	1.11E-04

Abbreviations: AF, allelic frequency; CI, confidence of intervals; gnomAD, genome aggregation database; OR, odds ratio; QGP, Qatar Genome Programme.
